# Altering Temporal Dynamics of Sleepiness and Mood During Sleep Deprivation: Evidence from Resting-State EEG Microstates

**DOI:** 10.3390/brainsci15040423

**Published:** 2025-04-21

**Authors:** Duo Bai, Xinrui Fan, Chuqin Xiang, Xu Lei

**Affiliations:** 1 Sleep and NeuroImaging Center, Faculty of Psychology, Southwest University, Chongqing 400715, China; 2 Key Laboratory of Cognition and Personality (Ministry of Education), Southwest University, Chongqing 400715, China

**Keywords:** sleep deprivation, EEG microstate, temporal dynamics, sleepiness, mood

## Abstract

**Objective:** Sleep deprivation negatively affects mood and sleepiness across subjective, objective, behavioral, and neuroimaging measures. However, the relationship between abnormal brain dynamics after sleep deprivation and mood or sleepiness impairments, from a temporal perspective, remains underexplored. Electroencephalogram microstate analysis offers a valuable approach for investigating the large-scale dynamics of brain networks. **Methods:** We implemented a strict sleep deprivation protocol with 71 participants, collecting resting-state electroencephalogram data, subjective sleepiness, objective alertness, and mood assessments after normal sleep and sleep deprivation (SD) conditions. Microstate time parameters were compared between sleep control (SC) and SD conditions. Additionally, we investigated how changes in these parameters correlated with subjective or objective measures of sleepiness and mood changes between SC and SD. **Results:** SD significantly decreased the mean duration and occurrence of microstate B while increasing those of microstate C. A decrease in microstate B occurrence significantly correlated with a higher Karolinska Sleepiness Scale score, whereas a reduction in microstate B duration indicated an increased response time on the Psychomotor Vigilance Performance. No significant associations were observed between microstate parameters and positive mood decline between SC and SD. Pearson correlation analysis was performed on the positive mood scores in both conditions. The findings demonstrated a significant positive relationship between positive mood scores and the mean duration of microstate B under SD conditions. **Conclusions:** Using a large SD dataset, this study demonstrated that subjective or objective sleepiness and positive mood were associated with decreased microstate B. These findings suggest that SD disrupts neural dynamics within the visual network.

## 1. Introduction

Sleep, a fundamental physiological process that occupies up to one-third of each day, is essential for human survival [1]. Sleep deprivation (SD) impairs performance and health [2]. However, SD is an effective experimental technique for understanding the relationship between sleep and various behavioral, emotional, and cognitive functions [3].

Previous studies on SD have examined the subjective and objective aspects of sleepiness and mood, yielding evidence ranging from behavioral to neuroimaging. Daytime sleepiness is the most widely utilized baseline measure for evaluating the effect of sleep disruption on cognitive function [4]. Subjective sleepiness is generally assessed using self-report scales such as the Karolinska Sleepiness Scale (KSS) and Stanford Sleepiness Scale, both of which have high reliability and validity [5,6]. These scales exhibit an inverse relationship between sleepiness scores and sleep duration, regardless of the number of nights of sleep restriction or deprivation [7]. The “gold standard” for evaluating the effect of SD on sleepiness behavior is the psychomotor vigilance task (PVT), a widely accepted measure of physiological sleepiness [8], particularly sensitive to prolonged wakefulness [9]. After SD, reaction times (RTs) typically increase along with the number of lapses [10].

Whether the neurophysiological basis of subjective and objective sleepiness consistently aligns remains uncertain. The two aspects of sleepiness––self-perception and self-behavior––are highly correlated [11]. These aspects are defined by combining subjective sleepiness and objective measures of mental alertness, enabling investigators to monitor the efficacy of sleep interventions [12]. Evidence from functional magnetic resonance imaging (fMRI) and resting-state electroencephalography (EEG) suggests that cortical activation patterns may be similar between increased subjective sleepiness and decreased PVT performance [13]. However, studies have reported inconsistent results regarding the independence and distinct neural mechanisms underlying the vulnerability of subjective sleepiness and objective vigilance to SD [14].

A meta-analysis revealed that SD is associated with a substantial reduction in positive mood, which is larger and more consistent than the increase in negative mood [15]. Neuroimaging evidence indicates that SD impairs mood by reducing the modularity of the salience network and shifting lateral mid-frontal connectivity dominance from the left to the right hemisphere [16]. Analysis of power envelope connectivity in resting EEG also demonstrated alterations in brain activity corresponding to mood changes. A decrease in positive mood has been linked to compromised integration between the posterior cingulate cortex and anterior medial prefrontal cortex [12].

Microstates refer to distinct patterns of brain activity observed in resting-state EEG, which reflect the brain’s ongoing network dynamics. From these microstates, a variety of parameters can be derived, providing insights into the temporal organization of brain network activity [17,18]. EEG microstates have been closely associated with resting-state networks identified through fMRI [19], including the default mode network (DMN) and the visual network. This connection is reinforced by combined EEG-fMRI and EEG source imaging studies [20]. Group spatial Independent Component Analysis (sICA) revealed that microstate A closely corresponds with blood oxygen level-dependent (BOLD) activation changes in the bilateral temporal lobes, whereas microstate B is associated with negative BOLD activation in the bilateral occipital cortex. Microstate C is linked to positive BOLD activation in the dorsal anterior cingulate cortex, bilateral inferior frontal cortex, and the right insular region. Microstate D is associated with negative BOLD activation in the dorsal and ventral regions of the right frontal and parietal cortices [20,21,22].

SD affects the functional connectivity of the primary visual cortex [23], directly impacting visual network activity, as revealed in resting-state fMRI studies involving patients with insomnia [24]. The impact of SD on the DMN varies. Krause et al. [25] reported that SD could decrease functional connectivity within DMN. However, other studies have suggested that abnormal activation in the DMN core region after SD is similar to that observed in individuals with insomnia [26,27].

To some extent, the spatial correspondence between EEG microstates and resting-state brain networks indicates their functional consistency. Recent meta-analyses have clarified the functional roles of microstates [19]. Microstate A is linked to auditory processing [21], whereas microstate B is associated with self-related visual processing, visualization, and cognition, including self-behavior and feelings [28]. Microstate C, rather than autonomic information processing, is associated with the processing of personally significant information, self-reflection, and self-referential internal mentation [19].

The microstates of sleepiness and emotions have some supporting data. A study performed a microstate analysis of resting-state EEG between sleep control (SC) and SD groups. The study included only 27 men; however, it discovered that subjective sleepiness was significantly negatively correlated with microstate A and positively correlated with microstate D [29]. Another study involving 30 male participants in a resting-state microstate analysis found that the impairment of objective vigilance induced by sleep deprivation was associated with changes in microstate B [30]. Chivu et al. [18] systematically reviewed EEG microstate changes in participants with mood disorders. Microstate B appeared more frequently, whereas microstate D occurred significantly less frequently in patients with mood disorders compared with healthy individuals. The characteristic parameters of microstates B and C contributed the most to the representation of positive and negative emotions [31].

Based on these findings, in this study, we aimed to use EEG microstate analysis on a larger sample to establish the relationship between abnormal brain dynamic activity and poor behavioral performance following SD. Additionally, we aimed to assess the temporal dynamics of resting EEG in SC and SD groups while evaluating participants’ demographics, subjective sleepiness, objective vigilance, and mood. We hypothesized that 1) SD would alter microstate B performance, which is associated with visual networks and 2) changes in microstate parameters would reflect shifts in subjective and objective sleepiness and mood, with mood alterations more strongly correlated with microstates B, C, and D.

## 2. Methods

### 2.1. Participants

Seventy-one participants provided informed consent in accordance with a protocol approved by the Southwest University Ethics Review Committee (Ethics Approval No. H20039). Participants were thoroughly briefed on the study’s objectives and procedures, and completed the informed consent form prior to the experiment. None of the participants reported mental illness, anxiety or depression symptoms, recent cold symptoms, or sleep-related problems such as insomnia, sudden wakefulness, or difficulty breathing. At the end of the experiment, additional financial compensation was offered to all participants. The current dataset is publicly available on OpenNeuro (https://openneuro.org/datasets/ds004902, accessed on 5 February 2025) [32].

### 2.2. Procedure

Participants were instructed to maintain regular daily work schedules and rest before the experiment. Sleep was monitored using a wristwatch (ActiGraph wGT3X-BT, Pensacola, FL, USA) and sleep diaries. Participants were asked to maintain their normal sleep patterns throughout the trial and avoid stimulant foods and drinks, such as coffee and alcohol, the day before the experiment. When the experiment commenced officially, all participants visited the laboratory twice. The two visits—one SD session and one SC session—were separated by approximately a week, and the participants’ order was randomized.

In the SD session, they were required to arrive at 9:00 p.m. and remain awake until the end of the experiment. Participants in the SD group underwent one night of continuous wakefulness while being monitored by a research assistant and a smart camera. They were forbidden to drink coffee, tea, food, or alcohol-related beverages. They could only engage in non-strenuous activities like drawing, reading, and conversation. They were prohibited from lying down, sleeping, or engaging in vigorous physical activity. The assistants would gently wake them up when they were about to sleep. In the SC session, participants were instructed to arrive at the laboratory after sleeping in the dormitory. Both sleep conditions were tracked using an actigraph and a sleep diary. The actigraph data were analyzed using ActiLife (https://www.theactigraph.com, accessed on 5 February 2025) to ensure that each participant had a regular sleep schedule or total SD for each condition.

Resting-state EEG recordings and questionnaires were administered in the morning. Participants first completed a 5 min PVT to assess objective sleepiness. Subsequently, they completed the KSS and the Positive and Negative Affect Scale (PANAS) to assess subjective levels of sleepiness and mood. Finally, EEG data were obtained between 9:00 and 10:00 a.m. Participants were instructed to keep their eyes open for 5 min while minimizing blinking and eye movements. For detailed experimental procedures and specific data, please refer to the original study [33].

### 2.3. Questionnaires and Behavior Measurement

Pittsburgh sleep quality index (PSQI): The PSQI is a self-reported assessment tool that measures sleep quality over 1 month, generating a global score and seven component scores. Subjective sleep quality, sleep latency, sleep duration, sleep efficiency, sleep disturbances, use of sleep medication, and daytime dysfunction comprise the component scores. The total score ranges from 0 to 21, with higher scores indicating poorer sleep quality [34]. Each component is scored on a scale of 0 to 3.

- Sleep diary: A sleep diary is a tool for self-evaluation of sleep [35]. Sleep diaries are simple to use and only require a few minutes a day to complete. It contains items such as “Time I went to bed last night” and “How alert did I feel when I got up this morning?”
- Subjective sleepiness: Subjective sleepiness was assessed using the KSS [36]. Participants chose one of nine descriptions to describe their current level of subjective sleepiness, ranging from s the first statement, “Extremely alert,” to the ninth statement, “Extremely sleepy, cannot keep awake.”
- Mood: Mood was evaluated using the PANAS, which includes 20 items describing emotional states, such as hostility. Each item is rated from 1 (nearly no) to 5 (extremely a lot) and grouped into two dimensions: negative affect (PANAS-NA) and positive affect (PANAS-PA).
- Objective sleepiness: The PVT was used to measure objective sleepiness [8]. The screen was centered on a square frame. Participants had to click on a mouse when the red number appeared in the box. The numbers reappeared after a random time. The PVT lasted for 5 min, with median RT and lapses (SC, RT > 500 ms; SD, RT > 700 ms) used to objectively measure vigilance.

### 2.4. EEG Data Acquisition and Data Preprocessing

We used 61 Ag/AgCl electrodes on an elastic cap to record EEG signals, following the 10–20 international electrode placement system (Brain Products GmbH, Steingrabenstr, Germany). All electrode impedance levels were kept below 5 kΩ, and electrooculograms in vertical and horizontal orientations were recorded using two channels. The FCz channel was used as the online reference channel at a sampling rate of 500 Hz. Six participants were excluded from the study because of excessive EEG artifacts. Consequently, data from 65 right-handed participants were included in the analysis.

Following the manual rejection of noisy data, an average of 225.13 ± 29.17 s of data in the SC condition and 231.00 ± 38.08 s in the SD condition was retained. For the bad electrodes, we interpolated the signal with the surrounding electrodes. Consequently, we interpolated 2.38 ± 2.15 electrodes in SC conditions and 2.44 ± 2.26 electrodes in SD conditions. In the SC and SD conditions, no significant differences were observed in the length of the valid data or the number of bad channels (paired *t*-test, *p* > 0.05).

Data preprocessing was performed using MATLAB R2021b (MathWorks) and EEGLAB (version 2021, http://sccn.ucsd.edu/, accessed on 5 February 2025). A general processing pipeline was used. Specifically, according to the latest guidelines for resting-state microstate analysis, appropriate bandpass filtering to the EEG signal before conducting microstate analysis (with a high-pass filter selected within the range of 1–2 Hz and a low-pass filter within the range of 20–40 Hz) is recommended. We applied band filtering within the 2–20 Hz range [37,38]. This process helps to preserve physiological brain signals while eliminating large amplitudes, low-frequency artifacts (such as sweating and skin potentials, etc.), and high-frequency artifacts (muscle and line noise, etc.).

Independent component analysis was employed to remove stereotyped muscle and ocular artifacts [39]. Finally, the signals were referenced to the average.

### 2.5. Microstate Analysis

The latest resting-state microstate analysis software MICROSTATELAB (https://sccn.ucsd.edu/eeglab/plugin_uploader/plugin_list_all.php, accessed on 11 September 2023) and MATLAB R2021b were used to analyze the clean continuous EEG data. The toolbox offers a full complement of tools for obtaining mean microstate maps across participants after optimizing their sequence for maximal shared variance, combining group mean template maps into grand mean template maps, visualizing the obtained results, comparing them to published templates, and updating the order of individual template maps based on a representative mean template [40].

Additionally, MICROSTATELAB allows the selection of microstate templates [41,42]. We discovered that, when applying 5–7 templates, no significant increase was observed in the global explained variance of all microstate classes or explained variance (EV) of any individual microstate class (EV of 4: SC, 54.6%; SD, 60.4% compared with EV of 5–7: SC, 56.6%; SD, 62.3%; SC, 57.6%; SD, 63.1%; SC, 58.9%; SD, 64.6%). We selected four canonically reported microstates to compare our findings with those of previous studies [42].

### 2.6. Statistical Analysis

Data analyses were performed using SPSS version 25.0. A paired-sample *t*-test was employed to compare the differences between SC and SD in the questionnaire and behavioral data. Regarding EEG data, alterations in the EEG microstate parameters were compared using a paired-sample *t*-test. Pearson’s correlation analyses were used to describe the relationships between variations in the questionnaire scores (KSS and PANAS: PANAS-PA and PANAS-NA), behavioral performance (PVT: RT and number of lapses), and alterations in microstate parameters during SC and SD. The significance level for all correlations was set at *p* < 0.05. To ensure the validity of the data quality, we performed an additional paired-sample *t*-test on the spectral characteristics of SC and SD, detailed in the Supplementary Materials.

## 3. Results

### 3.1. Sleep Deprivation on Subjective Sleepiness, Objective Vigilance, and Mood

Table 1 shows the descriptive statistics for participants’ demographic variables and behavioral metrics. A portion of the data was lost during the export process, resulting in different amounts of data for each section.

**Figure 1:**
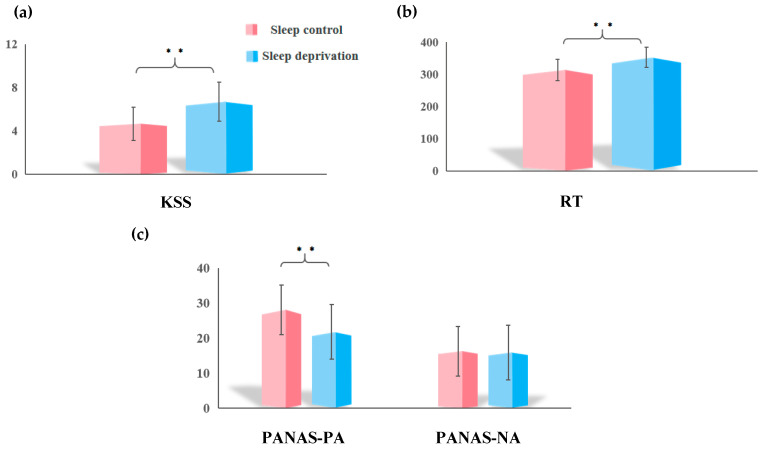
Differences between sleep control (SC) and sleep deprivation (SD) in terms of subjective and objective sleepiness and mood. (**a**) A paired *t*-test for the Karolinska Sleepiness Scale (KSS) indicated a significantly increased sleepiness in the SD group (*t* = −5.72, *p* < 0.001). (**b**) Psychomotor vigilance task (PVT) performance was evaluated using a paired *t*-test, and reaction time (RT) was increased in the SD group (*t* = −7.42, *p* < 0.001). (**c**) Paired *t*-tests demonstrated that Positive and Negative Affect Scale (PANAS)-Positive Affect (PA) scores decreased significantly in the SD group (*t*_PANAS-PA_ = 5.22, *p* < 0.001; *t*_PANAS-NA_ = 0.20, *p* = 0.840). ** *p* < 0.05, FDR-corrected.

A paired-sample *t*-test revealed a significant increase in KSS score and PVT RT after SD, as shown in Figure 1a (*t* = −5.72, *p* < 0.001) and Figure 1b (*t* = −7.42, *p* < 0.001). Regarding mood, no significant change was observed in the PANAS-NA scores. However, Figure 1c demonstrates a significant downward trend in PANAS-PA scores (*t*_PANAS-PA_ = 5.22, *p* < 0.001; *t*_PANAS-NA_ = 0.20, *p* = 0.840).

### 3.2. EEG Microstate Analysis

**Figure 2:**
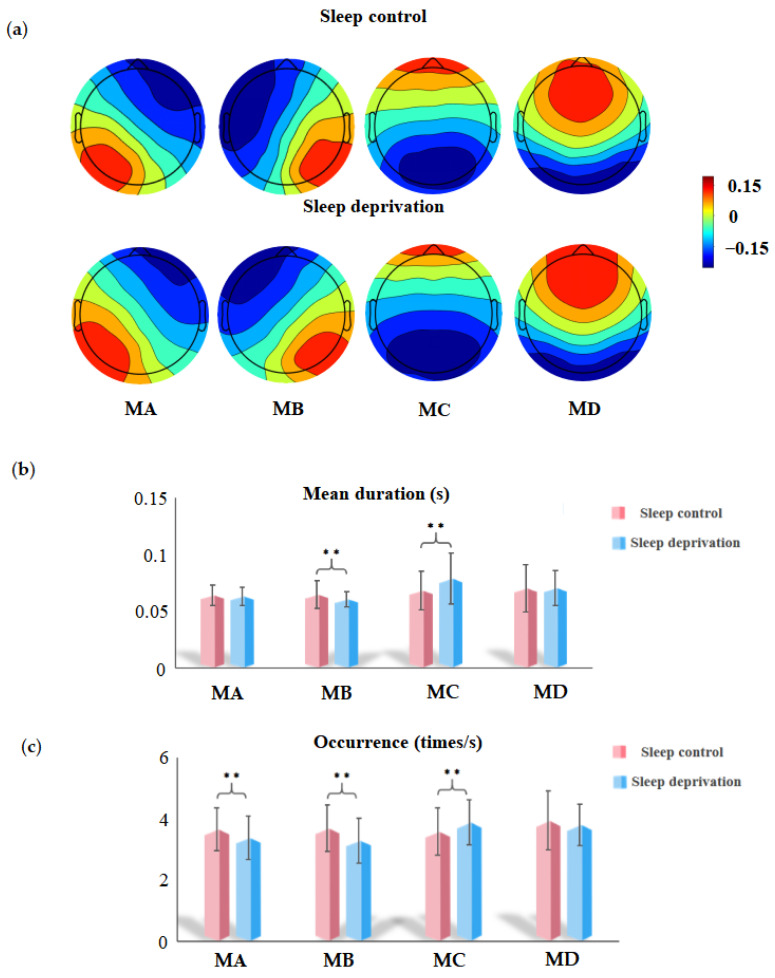
Spatial topography and parameters of microstate analysis between sleep control (SC) and sleep deprivation (SD). (**a**) Spatial configuration of the four classes of microstates in SC and SD (MA, microstate A; MB, microstate B; MC, microstate C; and MD, microstate D). (**b**) The paired *t*-test demonstrated that following SD compared with SC, the mean duration of class B decreased (*t*_B_ = 2.82, *p* < 0.01) and that of class C increased (*t*_C_ = −3.32, *p* < 0.01). (**c**) The paired *t*-test for occurrence revealed that class C had an increase in SD (*t*_C_ = −3.12, *p* < 0.01), and classes A and B had a significant decline (*t*_A_ = 2.77, *p* < 0.01; *t*_B_ = 3.42, *p* < 0.01). ** *p* < 0.05, FDR-corrected.

Figure 2a shows the topographic distributions of the SC and SD global maps. These spatial patterns are highly similar to those of the traditional templates found in earlier research. Figure 2b shows the results of the paired-sample *t*-test for the mean duration of the four microstate categories. Microstate C presents an increasing trend (*t*_C_ = −3.32, *p* < 0.01) following SD, but the mean duration of microstate B decreases (*t*_B_ = 2.82, *p* < 0.01) following total SD. Figure 2c illustrates the occurrences of microstates in the SC and SD states. After deprivation, the occurrence of microstate C significantly increased (*t*_C_ = −3.12, *p* < 0.01), whereas the occurrence of microstates A and B decreased (*t*_A_ = 2.77, *p* < 0.01; *t*_B_ = 3.42, *p* < 0.01). The time coverage of the microstate category after deprivation was similar to the occurrence; that is, the time coverage of microstates A and B decreased significantly (*t*_A_ = 2.65, *p <* 0.05; *t*_B_ = 3.79, *p* < 0.001), whereas that of state C increased significantly (*t*_C_ = −4.24, *p* < 0.001). Additionally, we have uploaded the SET file containing the grand mean as Supplementary Materials for online access.

**Figure 3:**
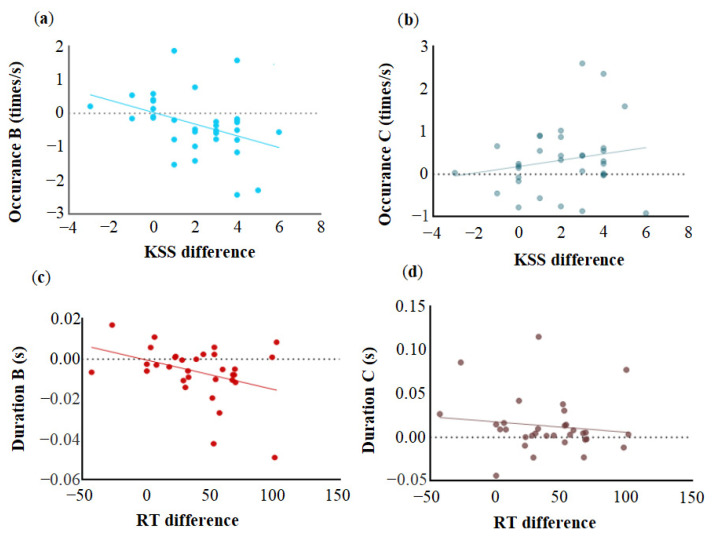
Microstates and subjective and objective sleepiness were assessed using Pearson analysis. (**a**) Subjective sleepiness was negatively correlated with the occurrence of microstate B (*r* = −0.40, *p* < 0.05) (**b**) but not with the occurrence of C (*r* = 0.19, *p* = 0.29). (**c**) Objective sleepiness was negatively correlated with the duration of microstate B (*r* = −0.37, *p* < 0.05) (**d**) but not with the duration of microstate C (*r* = −0.13, *p* = 0.48).

Pearson correlation analysis revealed an association between subjective and objective sleepiness, mood changes, and microstate alterations in the parameters caused by SD. Variations in subjective and objective sleepiness were defined as the differences between SD and SC scores, and alterations in microstate parameters were defined as the differences between SD and SC. As shown in Figure 3a, a significant negative correlation was observed between the change in subjective sleepiness and the occurrence of microstate B (*r* = −0.40, *p* < 0.05), but not with the occurrence of C (*r* = 0.19, *p* = 0.29) (Figure 3b). Similarly, Figure 3c shows that the difference in objective sleepiness was negatively correlated with the duration of microstate B (*r* = −0.37, *p* < 0.05), but not with microstate C duration (*r* = −0.13, *p* = 0.48) (Figure 3d).

**Figure 4:**
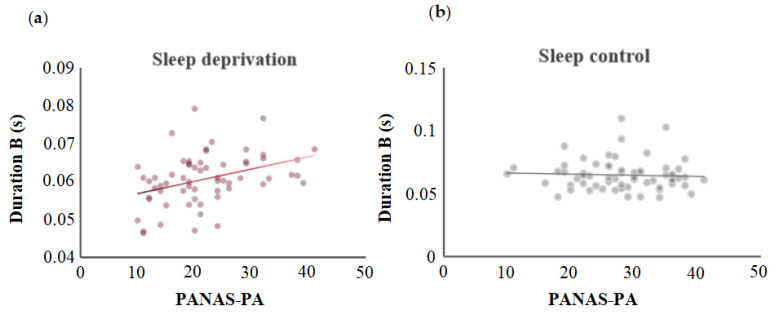
Pearson correlation analysis was performed on the characteristics of the microstates under sleep deprivation and control, as well as the PANAS scores under the corresponding states. (**a**) A significant positive correlation was observed between the mean duration of microstate B during sleep deprivation and PANAS-PA scores (*r* = 0.38, *p* < 0.01). (**b**) The duration of microstate B during sleep and the PANAS-PA scores exhibited no correlation (*r* = −0.05, *p* = 0.68).

Mood changes, as measured by the PANAS, were not correlated with differences in microstate parameters between the SC and SD conditions. However, Pearson correlation analysis between microstate performance and PANAS-PA scores during SD and normal sleep revealed a positive correlation between the mean duration of microstate B and PANAS-PA score after one night of SD (*r* = 0.38, *p* < 0.01) (Figure 4a). No correlation was observed between the duration of B under sleep conditions and the PANAS-PA dimension score after normal sleep (*r* = −0.05, *p* = 0.68) (Figure 4b). Additionally, a positive correlation was observed between the coverage of microstate B and PANAS-PA score after deprivation (*r* = 0.32, *p* < 0.01).

## 4. Discussion

This study, based on a large dataset, demonstrated an association between alterations in resting-state EEG microstates caused by SD and variations in behavior, considering subjective/objective sleepiness and mood. The sub-second temporal characteristic analysis revealed that the mean duration of microstate B significantly decreased after SD, whereas that of microstate C increased. The occurrence of microstates B and C showed a pattern similar to that of the mean duration. Concerning the relationship between deprivation-induced alterations in microstate characteristics and behavioral variations, we observed that changes in subjective sleepiness were negatively correlated with changes in B occurrence. Simultaneously, a negative correlation was observed between the variation in microstate B duration and RT. These findings suggest that insufficient sleep leads to alterations in characteristics associated with microstate B, which reflect both subjective sleep-related perceptions and objective behavioral performance. Further analysis of microstate parameters and positive mood scores in the SC and SD states demonstrated that the mean duration of microstate B after deprivation had a significant positive correlation with the PANAS-PA scores, suggesting that the impairment of positive mood caused by deprivation could be predicted, to some extent, by a decrease in the mean duration of microstate B.

### 4.1. Sleep Deprivation Altered Microstate B

Microstate analysis was performed on 65 participants, whose data were eventually retained using the most recent version of the standardized microstate analysis toolbox. Microstate B was significantly lower in the SD group compared to the SC group, which is consistent with the findings of Liu et al. [43]. Microstate B is primarily involved in the cuneus, right insula, and inferior and middle occipital gyri and has been linked to visual activity following simultaneous EEG-fMRI and source localization studies [18,41]. Research on SD effects on cognitive impairment indicates that acute SD can lead to significant alterations in the activity mechanisms and functional connectivity of visual networks [44,45]. Using the amplitude of low-frequency fluctuations and functional connectivity to investigate neurobiological changes caused by SD, researchers have discovered that increased amplitude of low-frequency fluctuations in the visual cortex and increased frontal–visual connectivity are associated with an increase in the number of PVT lapses [46]. Abnormal blood oxygen level-dependent activity and functional connectivity in the visual cortex may reflect deprivation-induced damage to the visual cortex. Patients with insomnia and those with chronic sleep problems reported abnormal node properties and weakened connections in the visual network, which may be related to negative mood in patients with insomnia or severe insomnia [47]. This finding aligns with our hypothesis that lack of sleep alters the mean duration and occurrence of microstate B, which in turn is related to changes in mood as well as subjective and objective measures of sleepiness.

Clinical trials on the treatment of depression have identified SD as a non-pharmacological treatment [48,49]. However, unmedicated patients with depression typically have a higher occurrence of microstate B [18], and the finding in this study that SD reduces the occurrence of microstate B actually supports the potential efficacy of sleep restriction as a treatment for depression.

The presence of microstate B positively relates to self-related feelings and behaviors [28]. Tarailis et al. [19] provided a systematic review of the functional role of microstate B, which has been associated with visual processing related to the self and self-visualization. This finding explains the significant correlation between the change in microstate B and the subjective perception and objective behavior of sleepiness in this study. A decrease in microstate B may be a probe for identifying individuals sensitive to SD.

Ke et al. [29] were the first to investigate the alterations in the temporal characteristics of specific brain states after SD using the resting-state EEG microstate technique. Using six topographic map templates, the authors discovered that SD significantly reduced the occurrence of microstate A sleep and was inversely related to subjective sleepiness on a visual analog scale. In their study, the occurrence of microstate D increased and was positively correlated with sleepiness scores. In our current study, microstate analysis was conducted within the framework of the four classical spatial modes of microstates (A, B, C, and D) [42,50,51,52] to establish neurophysiological interpretations concerning previous studies. After deprivation, a decrease in the occurrence of microstate A was observed; however, no increase was observed in the occurrence of microstate D. These contradictory findings may also be explained by the possible overlapping temporal dynamics between microstates C, D, and E when forcing the number of states to the canonical four microstates [18,19]. In this study, a higher KSS score was only found to be correlated with a decrease in the occurrence of microstate B, thus elucidating the relationships between microstate features of prototypical network types. Microstates A and B reinforce each other, indicating an interaction between auditory and visual sensory processing at rest [53].

Additionally, a connection between microstate B and objective sleepiness was identified. Baseline EEG activity before PVT predicts changes in RT between SC and SD, with occipital electrodes having the greatest predictive power [54]. According to an fMRI study, only the visual and sensorimotor networks revealed an interaction between sleep conditions and RT performance, indicating a close relationship between PVT RT changes and the visual network following SD [55]. Disruption of functional brain networks and loss of information acquisition and processing capacity after deprivation may be reflected in the increased PVT RT after SD [56,57]. Our finding that a short microstate B duration may indicate a decrease in visual processing ability, which occurs with longer RTs, supports this claim.

### 4.2. Sleep Deprivation Decreases Positive Mood

Despite the lack of a correlation between alterations in microstate B and changes in positive mood, we assumed that this might be attributed to mood state weakness and vulnerability [58]. The analysis conducted in different states revealed a positive correlation between the reduction in positive mood and the duration of microstate B under deprivation. In addition to the visual network, microstate B may be related to the salience network. Previous EEG-fMRI studies have demonstrated that microstate B involves the right insula, right claustrum, and other brain structures [29,41]. Studies on the effects of SD on resting brain activity have reported decreased spontaneous activity and functional connectivity in the insula [59,60,61]. The insula is a vital hub for integrating subjective feelings and emotional cognition [62,63]. Higher levels of positive mood are linked to increased functional connectivity between the insula and other brain regions [64]. After deprivation, a decrease was observed in insula activity, which was connected to mood and emotion [65,66]. This finding is consistent with the finding that a decrease in the duration of microstate B was significantly associated with a reduction in the positive dimension scores of the PANAS, which may indicate a decrease in insular activity. The reduction in the duration and occurrence of microstate B may serve as a potential marker for the effectiveness of depression treatment [18,67]. However, in the present study, the reduction in microstate B duration was associated with a decrease in positive mood, which seems contradictory. It is important to note that depressive symptoms are likely more closely related to the “negative affect” items on the PANAS [68,69,70]. Furthermore, depression is likely to have distinct subtypes, and sleep restriction may only be beneficial for certain subtypes [71].

Under normal sleep conditions, the duration of microstate B did not significantly correlate with positive mood scores. No correlation was observed between the two under normal sleep conditions, possibly because the mean durations of B and positive mood were high and reached a ceiling effect. Exploring the microstates associated with nine discrete emotions induced by video studies has demonstrated that the duration of microstate B does not characterize positive or negative emotions [31]. This finding justifies why no link between microstate B performance and positive mood was observed during sleep. Furthermore, mood and current emotional states are distinct, requiring more study.

### 4.3. Sleep Deprivation Altered Microstate C

The occurrence and mean duration of microstate C increased with deprivation. Similarly, resting-state microstate analysis following at least 18 h of sleep deprivation also confirmed this finding [72]. Studies examining the relationships between microstate features have demonstrated a competitive relationship between microstate C and other microstate types, suggesting that microstate C may play a unique role in prototypical microstate types [53]. Recent research on the relationship between resting brain networks and microstate categories has revealed that microstate C is primarily associated with the DMN [41,53]. The duration and occurrence of microstate C were significantly increased by applying transcranial magnetic stimulation to the DMN and specific regions of the dorsal attention network, such as the angular gyrus and intraparietal sulcus [73]. Research on the dynamic functional connectivity in the brain indicates that SD increases the within-network temporal variability of the DMN [26]. The increase in microstate C may be a compensatory mechanism for SD. However, as this study failed to observe a corresponding relationship with behavioral outcomes, further experimental data are needed to confirm these findings.

## 5. Limitations

First, we applied the classic four-microstate topographic map templates to describe our data. The global explained variance in our data by microstate categories was not improved by selecting more than five to seven templates. However, four microstates may limit the comparability of our findings to those of various previous studies. Future research should increase the number of participants and select more microstate templates based on the best interpretation rate for the data. Second, the interpretation of the functional significance of microstates remains subjective. Future studies should integrate experimental task paradigms to examine individual task performance following SD and validate the results of microstates B and C.

## 6. Conclusions

In this study involving a large sample of sleep-deprived individuals, we observed alterations in resting-state EEG microstates associated with behavioral impairments, particularly in subjective/objective sleepiness and mood changes. The decrease in microstate B was related to subjective/objective sleepiness and positive mood, suggesting impaired neural dynamics in the visual network caused by deprivation. The occurrence of microstate B was negatively correlated with changes in subjective sleepiness, whereas objective sleepiness was negatively correlated with the mean duration of microstate B. Under deprived conditions, a reduction in the mean duration of microstate B reflected a decreased positive mood. Conversely, the increase in microstate C following SD may serve as a compensatory mechanism, suggesting enhanced connectivity within the DMN. These findings deepen our understanding of the neurophysiological underpinnings of SD and its effects on brain dynamics and behavior, providing new insights into the role of microstates in sleep research.

## Figures and Tables

**Table 1:** Participants’ characteristics and psychological and behavior metrics.

	N_SC_ (N_SD_)	SC	SD	*t*	*p*
Age (year)	71	20.00 ± 1.44		-	-
Sex (female%)	71	47.89%		-	-
PSQI	66	5.05 ± 2.45		-	-
Diary	53 (51)	7.51 ± 1.42	-	-	-
KSS	33	4.67 ± 1.53	6.70 ± 1.81	−5.72	<0.001
PANAS-PA	71 (68)	27.96 ± 6.83	21.72 ± 7.96	5.22	<0.001
PANAS-NA	71 (68)	16.73 ± 5.99	16.63 ± 6.23	0.20	0.840
Median RT (RT, ms)	38	315.54 ± 32.04	355.26 ± 32.73	−7.42	<0.001
Number of lapses (times)	38	2.00 ± 2.37	2.24 ± 2.75	−0.25	0.808
Standard deviation of RT	38	58.27 ± 16.54	78.55 ± 18.61	−4.27	<0.001

Note: Data are presented as mean ± standard deviation. N_SC_ (N_SD_) is the number of valid data points for sleep control (along with the number of valid data points for sleep deprivation). SC, sleep control; SD, sleep deprivation; PSQI, Pittsburgh Sleep Quality Index; Diary, sleep quality of last night sleep (1–10); KSS, Karolinska Sleepiness Scale; Positive and Negative Affect Scale, PANAS-PA: positive affect; PANAS-NA: negative affect; RT, reaction time.

## Data Availability

The available data from the current study can be accessed on OpenNeuro (https://openneuro.org/datasets/ds004902, accessed on 5 February 2025).

## Supplementary Materials

The following supporting information can be downloaded at: https://www.mdpi.com/article/10.3390/brainsci15040423/s1, File S1: Supplemental Material.
