# Supplementary material for: Altering Temporal Dynamics of Sleepiness and Mood During Sleep Deprivation: Evidence from Resting-State EEG Microstates
Source: Brain Sci. 2025 Apr 21;15(4):423. doi: 10.3390/brainsci15040423 (PMC12025901; doi:10.3390/brainsci15040423)
Supplement: Supplementary file 1 [file brainsci-15-00423-s001.zip › brainsci-3557146-supplementary.pdf]

Supplement Material

To ensure the validity of the experimental procedure, we additionally performed frequency spectrum analysis on the resting-state EEG data between sleep deprivation (SD) and sleep control (SC). The EEG preprocessing pipeline closely followed the procedures described for microstate analysis in the main text, with the exception that the filtering range was adjusted to 0.2-45Hz. The frequency spectrum was calculated using the Welch method.The absolute power for each electrode was logarithmically transformed to calculate the power spectrum (1 dB = 10 × log(μV^2)). The power spectrum data for the Cz electrode were averaged, and the mean and standard deviation for each participant were calculated under the conditions of sleep control (SC) and sleep deprivation (SD). Based on Figure S1, it can be observed that following sleep deprivation, there was an increase in theta (4-8 Hz) energy across all frequency bands. Our findings are in agreement with previous results(Del et al.,2019; Tramonti et al.,2022; Lian et al.,2023).

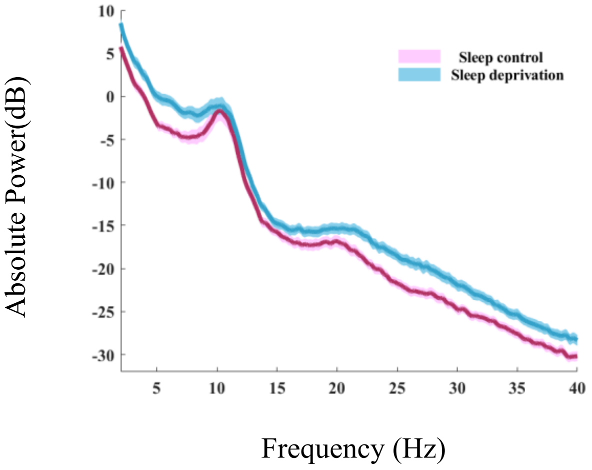

Figure S1. EEG power spectrum during sleep control (pink) and sleep deprivation (blue).

The topographical maps of theta (4-8 Hz) for Sleep Deprivation (SD) and Sleep control (SC) are shown in the Figure S2. After sleep deprivation, there is an increase in the energy of theta ($t = -26.23$, $p < 0.000$).

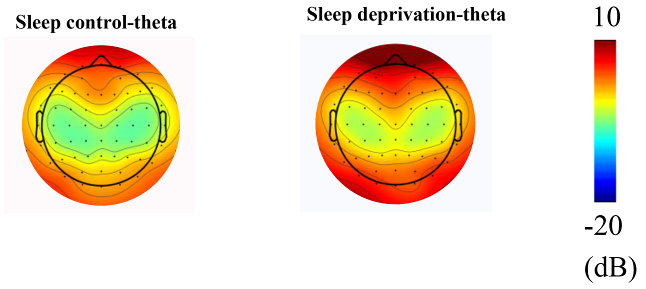

Figure S2 Topography of resting-state EEG in theta for sleep control and sleep deprivation conditions.

Reference:

Del Percio, C., Derambure, P., Noce, G., Lizio, R., Bartrés-Faz, D., Blin, O., Payoux, P., Deplanque, D., Méligne, D., Chauveau, N., Bourriez, J. L., Casse-Perrot, C., Lanteaume, L., Thalamas, C., Dukart, J., Ferri, R., Pascarelli, M. T., Richardson, J. C., Bordet, R., Babiloni, C., ⋯ PharmaCog Consortium (2019). Sleep deprivation and Modafinil affect cortical sources of resting state electroencephalographic rhythms in healthy young adults. Clinical neurophysiology : official journal of the International Federation of Clinical Neurophysiology, 130(9), 1488–1498. https://doi.org/10.1016/j.clinph.2019.06.007

Lian, J., Xu, L., Song, T., Peng, Z., Zhang, Z., An, X., Chen, S., Zhong, X., & Shao, Y. (2023). Reduced Resting-State EEG Power Spectra and Functional Connectivity after 24 and 36 Hours of Sleep Deprivation. Brain sciences, 13(6), 949. https://doi.org/10.3390/brainsci13060949

Tramonti Fantozzi MP, Banfi T, Di Galante M, Ciuti G, Faraguna U. Sleep Deprivation-Induced Changes in Baseline Brain Activity and Vigilant Attention Performance. Brain Sci. 2022 Dec 9;12(12):1690. doi: 10.3390/brainsci12121690. PMID: 36552150; PMCID: PMC9775863.
